# Crosstalk between exosomes and autophagy: A review of molecular mechanisms and therapies

**DOI:** 10.1111/jcmm.16276

**Published:** 2021-01-27

**Authors:** Huifang Xing, Jin Tan, Yuyang Miao, Yingmei Lv, Qiang Zhang

**Affiliations:** ^1^ Department of Geriatrics Tianjin Medical University General Hospital Tianjin Geriatrics Institute Tianjin China; ^2^ Tianjin Medical University Tianjin China

**Keywords:** autophagy, exosomes, mesenchymal stem cells, miRNA, molecular mechanisms, therapies

## Abstract

Exosomes are extracellular vesicles that primarily exist in bodily fluids such as blood. Autophagy is an intracellular degradation process, which, along with exosomes, can significantly influence human health and has therefore attracted considerable attention in recent years. Exosomes have been shown to regulate the intracellular autophagic process, which, in turn, affects the circulating exosomes. However, crosstalk between exosomal and autophagic pathways is highly complex, depends primarily on the environment, and varies greatly in different diseases. In addition, studies have demonstrated that exosomes, from specific cell, can mitigate several diseases by regulating autophagy, which can also affect the excessive release of some harmful exosomes. This phenomenon lays a theoretical foundation for the improvement of many diseases. Herein, we review the mechanisms and clinical significance of the association and regulation of exosomes and autophagy, in order to provide a new perspective for the prevention and treatment of associated diseases.

## 1 INTRODUCTION

An exosome is an extracellular vesicle with a diameter of 30‐100 nm that naturally exists in bodily fluids, including blood, saliva, urine and breast milk. In 1983, Pan et al^1^ had observed that, in sheep, immature red blood cells can secrete vesicles, which were later referred to as ‘exomes’ by Johnstone et al^2^ in 1987. Studies have since reported that various cells can secrete exosomes, which were considered cellular waste. In 1996, Stoorvogel et al^3^ illustrated that exosomes could change the extracellular microenvironment, participate in immune regulation, and affect overall health, but the mechanisms remained unclear. In 2007, Valadi et al^4^ found that genetic information could be exchanged between cells via exosomes; further investigations had identified more exosomal components, such as lipids, proteins, mRNA and miRNA that participate in the body's immune responses, antigen presentation, cell migration and differentiation, tumour invasion and autophagy.^5^ Coordination between exosomes and autophagy plays an important role in maintaining intracellular homeostasis.^6^, ^7^

Autophagy is a common metabolic process in most eukaryotic cells, functioning to promote cell survival.^8^ Under various stress signals, such as starvation, hypoxia or endoplasmic reticulum stress, autophagy can degrade soluble proteins and other organelles into amino acids in the cytoplasm for energy production and biosynthesis as a self‐protective mechanism. In addition, autophagy clears denatured or misfolded proteins, and ageing or damaged organelles to maintain intracellular homeostasis.^9^ Under severe or chronic stress, excessive or insufficient autophagy can lead to the accumulation of a large amount of self‐degradation or toxic substances, ultimately leading to cell death, which is closely associated with the pathogenesis of various cancers, as well as neurodegenerative, metabolic‐related and immune diseases.^10^, ^11^ Recent studies have shown that stress and pathological conditions regulate autophagy through exosomes and their cargos. For example, under hypoxic conditions, exosomes released by cardiomyocytes transferred miR‐30a to adjacent cells, thereby inhibiting autophagy by interrupting the Beclin‐1 pathway, which reduces myocardial injury.^12^ Exosomes secreted by breast cancer cells transferred miR‐126 to adipocytes, which induced autophagy through the AMPK/mTOR pathway, thereby altering adipocyte metabolism and promoting cancer progression.^13^ Conversely, insufficient or excessive autophagy affects the release of exosomes. For example, insufficient autophagy increases the amount of exosomes, which promotes the diffusion of α‐synuclein and exacerbates the progression of Parkinson's disease (PD).^14^ Therefore, the relationship between exosomes and autophagy warrants further investigation.

An increasing number of studies have demonstrated that exosomal and autophagic pathways are cross‐regulated and affect the development of various diseases. This article reviews the following four findings to provide a new perspective for the prevention and treatment of related diseases: (a) exosomes, and their cargos, can regulate autophagic pathways via different mechanisms, which is highly dependent on the environment and cell source; (b) exosomes derived from certain cells, especially mesenchymal stem cells (MSCs), can mitigate several diseases by regulating autophagy; (c) autophagy can affect the formation and release of exosomes; (d) intervening in the key signalling pathways and molecules of autophagy can reduce the release of harmful exosomes, thereby relieving the symptoms of several diseases.

## 2 REGULATION OF AUTOPHAGY BY EXOSOMES

### 2.1 Components and trafficking of exosomes

**FIGURE 1:**
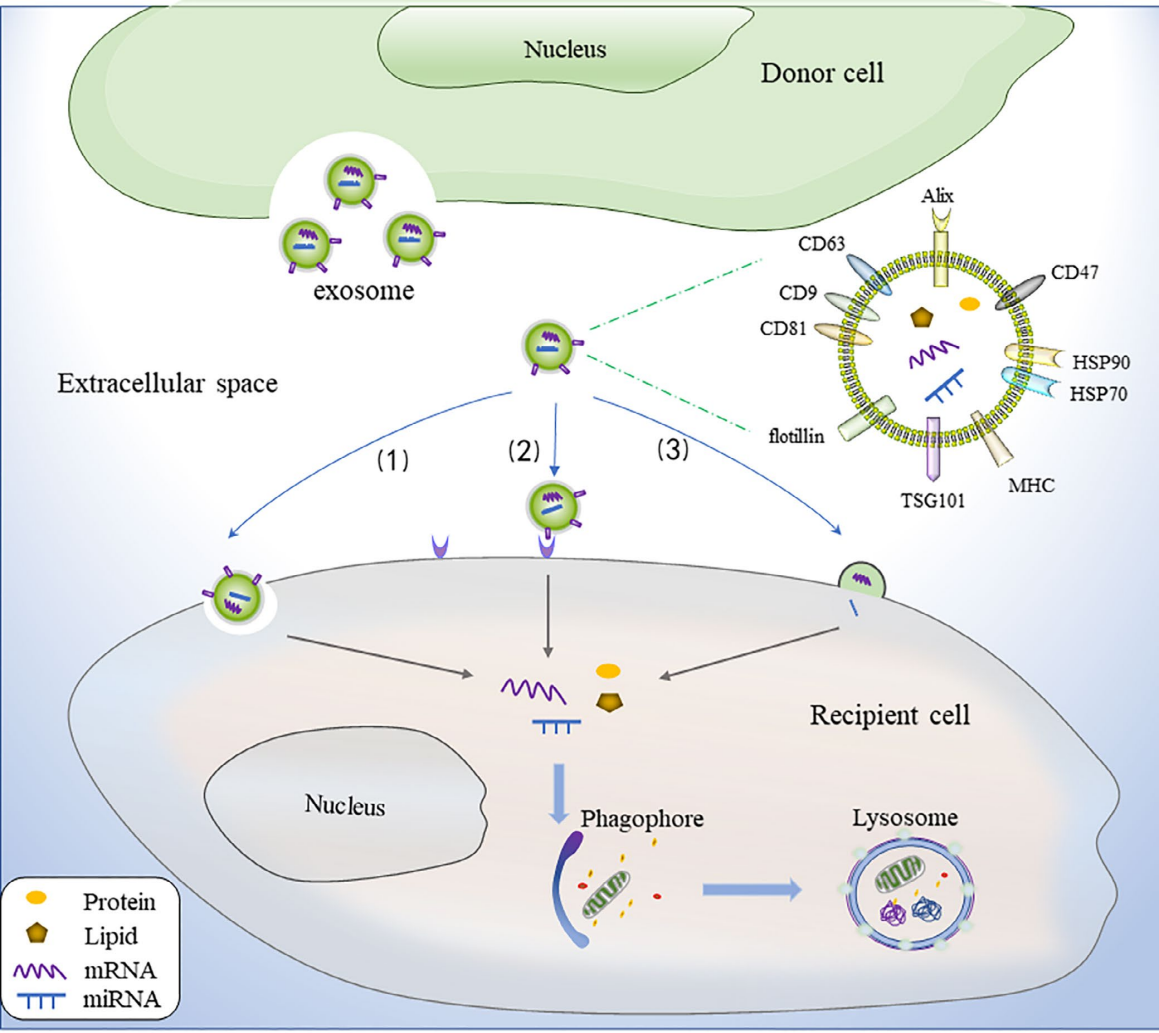
Components of exosomes and trafficking. Exosomes are secreted into the extracellular space by donor cells and have commonly conserved components, including CD9, CD63, CD81, Alix, flotillin, TSG101, MHC, HSP70, HSP90 and CD47. Exosomes can carry various cargos and interact with recipient cells primarily via three pathways: (1) phagocytosis, (2) ligand‐receptor binding and (3) membrane fusion. Following uptake by recipient cells, exosomes release their cargo, which can modulate autophagy

Exosomes are small vesicles composed of lipid bilayer membranes that contain proteins, nucleic acids, lipids and other substances. They have conserved components, including tetraspanin proteins (CD9, CD63, CD81), Alix, flotillin, TSG101, immunomodulatory proteins (MHC), heat shock proteins (HSP70, HSP90), and CD47^15^ (Figure 1). CD9, CD81, CD63, flotillin, TSG101 and Alix, which are exosomes biomarkers, are involved in biogenesis, cargos clustering, and exosomes secretion.^5^ MHC controls the exchange of antigen information between immune cells.^5^ HSP70 and HSP90 help exosomes adapt to the extracellular environment^16^; CD47 on exosomes produces a ‘don't eat me’ signal, preventing exosomes from being digested by monocytes and macrophages, thus improving their stability in the body.^17^ Apart from conserved proteins, exosomes also express cell‐specific proteins that reflect the origin of donor cells. For example, exosomes derived from platelets contain von Willebrand factor and integrin CD41a, whereas T cell‐derived exosomes contain CD3.^18^, ^19^ Exosomes thus characterize the origin of parental cells and share some of their functional characteristics. In addition, exosomes carry various cargos, such as mRNAs, miRNAs and siRNAs, which can be transferred to recipient cells and affect the expression of the corresponding genes, several of which are associated with autophagic proteins^20^ (Figure 1). However, the type and amount of exosomal cargo depends on the physiological or pathophysiological conditions of the donor cells. For example, hypoxia induced the upregulation of miR‐30a in myocardial cell‐derived exosomes,^12^ whereas cigarette smoke increased exosomal miR‐210 in bronchial epithelial cells.^21^ Exosomal miR‐7‐5p has also been observed to increase in irradiated cells.^22^

Exosomes interact with recipient cells primarily via three pathways^23^, ^24^: (a) phagocytosis; (b) ligand‐receptor binding; and (c) membrane fusion (Figure 1). These mechanisms may occur solely or in combination, and the mechanisms may change under different stress conditions, which requires further investigations. In the basic experiment, exosomes can be isolated and purified from the cultured cell supernatant, and then injected intravenously into study subjects such as mice, or added directly to the cell medium for uptake.^25^, ^26^

### 2.2 Molecular mechanisms and biological effects of autophagy

**FIGURE 2:**
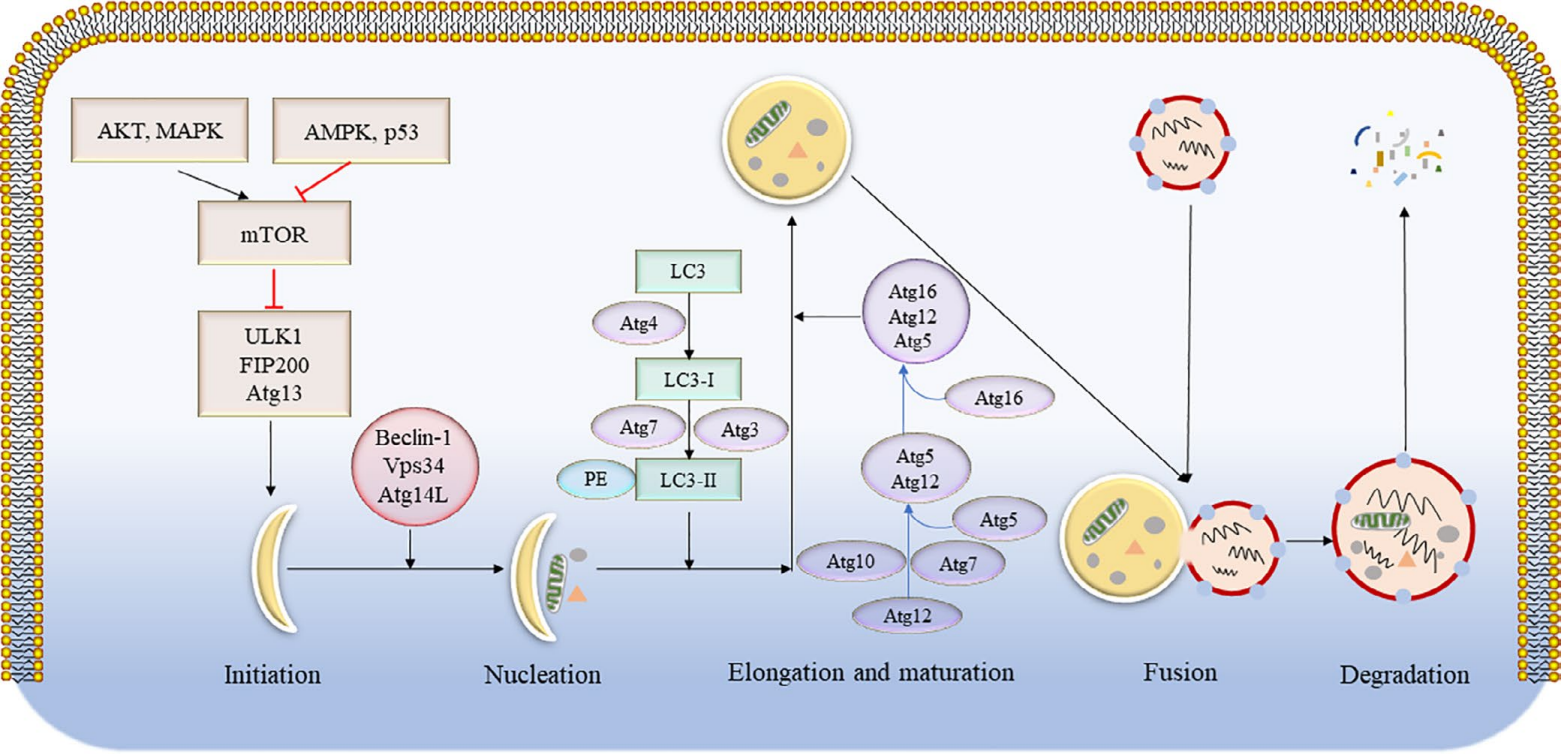
Schematic diagram of the molecular mechanism of autophagy. The autophagic process includes five primary stages, namely initiation, nucleation, elongation and maturation, fusion and degradation. The molecular pathway, comprising the core autophagy proteins, is illustrated. PE, phosphatidylethanolamine

The autophagic process includes five primary stages: initiation, nucleation, elongation and maturation, fusion and degradation.^27^ The mammalian target of rapamycin (mTOR) is the key relator of the initiation stage, in which its activation (ie Akt and MAPK signals) inhibits autophagy, whereas its negative regulation (ie AMPK and P53 signals) induces autophagy. Under stress conditions, mTOR is inactivated, whereas the ULK complex (composed of ULK1, FIP200, and autophagy‐related protein 13 [Atg13]) is activated.^28^ Beclin‐1 is an important molecule for autophagosome formation,^29^ which then forms a complex with Vps34 and Atg14L, promoting the nucleation stage and recruiting proteins from the cytoplasm.^30^, ^31^ In the elongation and maturation stage, two ubiquitin‐like conjugation systems are required to promote the extension of the autophagosome membrane. The first system involves the microtubule‐associated protein light chain 3(LC3)‐phosphatidylethanolamine (PE) complex. LC3 is cleaved by Atg4 at its C terminal to produce intracellular LC3‐I, which is conjugated with PE in the ubiquitin‐like reactions of Atg7 and Atg3. The lipid form of LC3 (LC3‐II) is attached to the autophagosome membrane.^32^ The second system involves the Atg12‐Atg5‐Atg16 complex, in which Atg12 is conjugated with Atg5 via ubiquitin‐like reactions of Atg7 and Atg10. The Atg12‐Atg5 conjugate interacts noncovalently with Atg16 to form a large complex.^33^ In the fusion stage, autophagosomes and lysosomes fuse to form autolysosomes, whereas in the degradation stage, cargos inside the autolysosomes are degraded (Figure 2).

Autophagy is strictly regulated to maintain homeostasis. Once autophagy is initiated, multiple Atg proteins cooperate to coordinate the next steps of autophagy. Whether autophagy imparts a protective function in diseases remains debatable.^34^ For instance, insufficient autophagy contributes to the accumulation of tau and synuclein proteins, and promotes neurodegenerative disease.^35^ In the context of cancer, autophagy has been shown to initially act as a tumour suppressant but later protecting tumour cells from the immune system's defence mechanisms.^36^ Similarly, heart and liver diseases have been shown to be positively and negatively regulated by autophagy, respectively. Therefore, the regulation of autophagy by exosomes is complex and may produce different (or even opposite) effects in various diseases.

### 2.3 Related signalling pathways in autophagic regulation by exosomes

**FIGURE 3:**
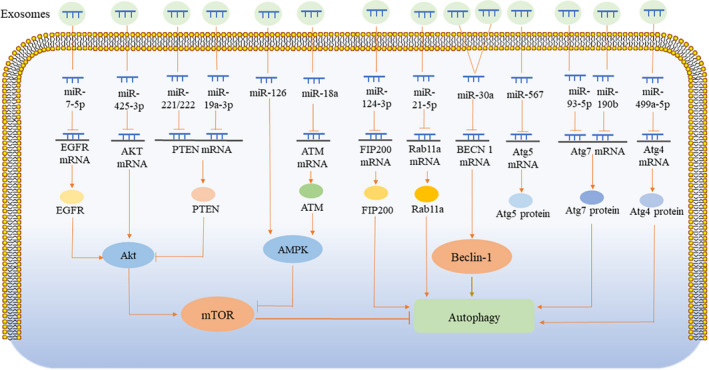
Schematic summary of the related signalling pathways in the context of exosomes‐mediated autophagy regulation. Exosomes release their cargos into recipient cells that can regulate autophagy. The different effects of exosomes on autophagy regulation and the different signalling pathways affected, primarily due to the different cargos they carry. Exosomal miRNA recognizes its target mRNA, suppresses the translation of target mRNA, and reduces related proteins. The synthesis of mTOR, Beclin‐1, Atgs, and their upstream proteins, are blocked, which in turn affects the process of autophagy

As mentioned above, autophagy involves a complex set of molecules and proteins, and abnormalities in any one gene or protein can affect the level of autophagy. Among these, mTOR and Beclin‐1 are the intersection points of multiple signalling pathways and key factors in autophagic regulation. The expression levels of these proteins can affect the autophagic level of target cells. As an important exosomal cargo, miRNAs are endogenous non‐coding RNAs with regulatory functions that can degrade target mRNAs. Each miRNA can have multiple target genes, whereas several miRNAs can also regulate the same mRNA. Exosomes from different cell sources rich in different miRNAs and molecules under different pathological conditions, as well as their regulatory autophagic pathways, vary greatly (Table 1 and Figure 3).

**TABLE 1:** Related signalling pathways in autophagic regulation by exosomes

Condition	Donor cell	Exosome cargos	Recipient cell	Signalling pathway	Autophagy	Effect	Reference
Cisplatin	NSCLC cells	miR‐425‐3p↑	A549 cells	Akt/mTOR	Induce	Deleterious	[37]
Radiation	BEP2D cells	miR‐7‐5p↑	Unirradiated BEP2D cells	EGFR/Akt/mTOR	Induce	Deleterious	[20]
Atherosclerosis	HAoSMCs	miR‐221/222↑	HUVECs	PTEN/Akt/mTOR	Inhibit	Deleterious	[39]
Parkinson	SH‐SY5Y cells	miR‐19a‐3p↑	Microglias	PTEN/Akt/mTOR	Inhibit	Deleterious	[40]
Breast cancer	breast cancer cells	miR‐126↑	Adipocytes	AMPK/mTOR	Induce	Deleterious	[12]
Mtb‐infected	RAW264.7 cell	miR‐18a↑	Macrophages	ATM/AMPK/mTOR	Inhibit	Deleterious	[41]
Hypoxia	H9c2 cells	miR‐30a↑	Adjacent H9c2 cells	Beclin‐1	Inhibit	Deleterious	[11]
AMI	H9c2 cells	miR‐30a↓	Adjacent H9c2 cells	Beclin‐1	Induce	Deleterious	[46]
MIRI	Myocardial cells	miR‐30a↓	Adjacent myocardial cells	Beclin‐1	Induce	Deleterious	[47]
Hepatic fibrosis	LX‐2 cells	miR‐30a↓	Adjacent LX‐2 cells	Beclin‐1	Induce	Deleterious	[48]
AMI	ADSCs	miR‐93‐5p↑	Cardiomyocytes	Atg7	Inhibit	Beneficial	[49]
OGD	Microglias	miR‐190b↑	Neurons	Atg7	Inhibit	Beneficial	[50]
Breast cancer	None^a^	miR‐567↑	SKBR‐3 cells	Atg5	Inhibit	Beneficial	[51]
Synovitis	Chondrocytes	miR‐449a‐5p↑	Macrophages	Atg4	Inhibit	Deleterious	[52]
TBI	Neurons	miR‐21‐5p↑	Adjacent neurons	Rab11a	Inhibit	Beneficial	[53]
TBI	Microglias	miR‐124‐3p↑	Neurons	FIP200	Inhibit	Beneficial	[54]

Abbreviations: A549, human NSCLC cell line; ADSCs, adipose‐derived stromal cells; AMI, acute myocardial infarction; ATM, ataxia telangiectasia mutated; BEP2D, human bronchial epithelial cell line; H9c2, cardio‐myoblasts cell line; HAoSMCs, human aortic smooth muscle cells; HUVECs, human umbilical vein endothelial cells; LX‐2, immortalized hepatic stellate cells; MIRI, myocardial ischaemia‐reperfusion injury; Mtb, *Mycobacterium tuberculosis*; NSCLC, non‐small cell lung cancer; OGD, oxygen glucose deprivation; RAW264.7, mouse mononuclear macrophage leukaemia cells; SH‐SY5Y, human neuroblastoma cells; SKBR‐3 cells, Human HER‐2‐positive breast cancer cell lines; TBI, traumatic brain injury.

^a^ miR‐567 was packed into exosomes manually.

#### 2.3.1 mTOR signalling pathway

mTOR is an evolutionarily conserved serine/threonine protein kinase that belongs to the phosphatidylinositol‐3 kinase (PI3K) protein kinase family. The mTOR pathway is the cross point of several signalling pathways, and is the primary signalling pathway in autophagic regulation. Its upstream components include the Akt/mTOR and AMPK/mTOR pathways.^37^

Akt is a serine/threonine specific protein kinase that inhibits autophagy by activating mTOR. Akt1 is reportedly the direct target gene of miR‐425‐3p.^38^ Ma et al^39^ reported that higher expression of exosomal miR‐425‐3p was induced upon cisplatin treatment of human non‐small cell lung cancer cells. In A549 cells, exosomes can induce autophagy by targeting the Akt/mTOR pathway, resulting in chemotherapy resistance of the target cells. Song et al^22^ found that EGFR, a receptor tyrosine kinase on the upstream of Akt/mTOR, was the direct target of miR‐7‐5p. Exosomal miR‐7‐5p secreted by human bronchial epithelial cells, BEP2D, upregulated following radiation and transferred to unirradiated BEP2D. This inhibited the expression of EGFR, which, in turn, inhibited the phosphorylation of Akt and mTOR and induced the autophagy of unirradiated BEP2D, thereby causing cell damage.^22^ Li et al^40^ found that PTEN, a tumour suppressor gene that can inhibit Akt's activity, was a target gene of miR‐221/222. During cardiovascular dysfunction and atherosclerosis, miR‐221/222 is upregulated in human aortic smooth muscle cell‐derived exosomes, which can inhibit the autophagy of human umbilical vein endothelial cells, leading to apoptosis via targeting of the PTEN/Akt/mTOR signalling pathway. Li et al^41^ established a Parkinson's model by transfecting α‐synuclein into human neuroblastoma cells (SH‐SY5Y). Exosomes derived from these cells expressed high levels of miR‐19a‐3p, which inhibits the autophagy of recipient microglia by regulating the PTEN/AKT/mTOR signalling pathway, thus weakening the intracellular degradation of harmful α‐synuclein. Wu et al^13^ found that breast cancer cells co‐cultured with adipocytes showed increased expression of miR‐126, which was subsequently released by exosomes. MiR‐126 promotes cancer cell metastasis by inducing AMPK phosphorylation and activating autophagy. ATM, a protein kinase, is upstream of AMPK and a potential target for miR‐18a. Yuan et al^42^ discovered that the expression of miR‐18a was gradually upregulated in a time‐dependent manner in exosomes derived from mouse mononuclear macrophage leukaemia cells (RAW264.7 cells) infected with *Mycobacterium tuberculosis*. MiR‐18a inhibited macrophage autophagy in cells infected by *M. tuberculosis* by regulating the ATM‐AMPK autophagy pathway.^42^

#### 2.3.2 Beclin‐1 signalling pathway

Beclin‐1, a vital protein in the autophagic process, comprises three prominent domains: Bcl‐2‐homology‐3 (BH3), coiled coil (CCD) and evolutionarily conserved (ECD) that contains important functional sites.^43^ Several autophagic regulatory proteins bind to the different domains of Beclin‐1 to form protein complexes that regulate autophagic levels. III‐type phosphoinositide 3‐kinase (Class III PI3K, also known as Vps34) combines with Beclin‐1 at the ECD site, recruiting other autophagic regulatory proteins such as Atg14 in the cytoplasm to form protein complexes, which promotes the extension of the lipid membrane and the maturation of autophagosomes.^44^, ^45^ However, Bcl‐2, an inhibitor of apoptosis, binds to Beclin‐1 at the BH3 site and inhibits the formation of these complexes, thereby inhibiting Beclin‐1‐dependent autophagy.^45^

Beclin‐1 is encoded by BECN 1, which is the target gene of miR‐30a.^46^ Exosomal miR‐30a inhibits autophagy by targeting the Beclin‐1 signalling pathway and plays an important role in myocardial infarction (MI) and liver fibrosis. Yang et al^12^ found that exosomal miR‐30a was released by hypoxic cardiomyocytes and transferred between cardiomyocytes, resulting in cardiomyocyte injury by inhibiting autophagy. Meanwhile, inhibitors of both exosomes and miR‐30a can increase autophagy and reduce cardiomyocyte injury. Inconsistent with Yang et al's findings, Zhang et al^47^ demonstrated that epigallocatechin gallate, a myocardial injury protective agent, upregulated exosomal miR‐30a to inhibit autophagy and apoptosis in acute MI (AMI). Xu et al^48^ also found that exosomal miR‐30a reduced cardiomyocyte apoptosis by inhibiting autophagy in rats with myocardial ischaemia/reperfusion (I/R) injury, which was not consistent with Yang's results, either. Autophagy is acutely activated during hypoxia, where it elicits a protective response, thereby mediating cellular adaptation and survival. However, with the prolongation of myocardial hypoxia, excessive autophagy can lead to the accumulation of a large amount of self‐degradation or toxic substances, ultimately leading to cell death. In Yang et al's study, exosomal miR‐30a contributed to the inhibition of autophagy; therefore, the protective autophagy in cardiomyocytes following hypoxia was insufficient, which resulted in the apoptosis of cardiomyocytes. In the myocardial injury models established by Zhang and Xu, excessive autophagy damaged cardiomyocytes. Exosomal miR‐30a reduced cardiomyocyte apoptosis by inhibiting excessive autophagy. In addition, excessive autophagy contributed to liver fibrosis. Chen et al^49^ established a model of hepatic fibrosis induced by TGF‐β1, in which exosomal miR‐30a secreted by hepatic stellate cells was downregulated. The overexpression of miR‐30a may alleviate liver fibrosis by inhibiting autophagy mediated by the Beclin‐1 signalling pathway.

#### 2.3.3 Other signalling pathways

Other molecules, such as a variety of Atgs, Rab11a, and FIP200, are essential during the autophagic process. Liu et al^50^ demonstrated that exosomal miR‐93‐5p inhibited excessive autophagy by targeting Atg7 in AMI, whereas Pei et al^51^ found that exosomes released by astrocytes inhibited neuronal apoptosis under the condition of ischaemia/hypoxia. This is because the exosomes secreted by the astrocytes transferred miR‐190b to neurons and reduced apoptosis by targeting Atg7 to inhibit autophagy. Han et al^52^ illustrated that miR‐567‐packed can bind to exosomes, inhibit autophagy, and reverses chemotherapy resistance by targeting Atg5. Ni et al^53^ found that miR‐449a‐5p‐rich exosomes are secreted by chondrocytes during osteoarthritis, which could enter macrophages and inhibit autophagy by targeting Atg4, further aggravating synovitis and cartilage erosion. During traumatic brain injury (TBI), the expression of exosomal miR‐21‐5p is also increased, which inhibits neuronal autophagy by targeting Rab11a, thereby alleviating autophagy‐mediated nerve injury.^54^ Increased levels of miR‐124‐3p in microglial exosomes following TBI have been shown to play a neuroprotective role by targeting FIP200‐mediated neuronal autophagy.^55^ In addition, exosomes derived from microglia inhibited autophagy in ischaemic stroke and protected against neuronal damage, although the mechanism remains unclear.^56^

In short, exosomes and their contents, primarily miRNAs, can regulate the autophagic level of target cells via mTOR, Beclin‐1, Atgs and other signalling pathways. Although research on how exosomes and their cargo participate in autophagic regulation remains in its preliminary stage, several recent studies have found that exosomes derived from MSCs have therapeutic potential via the regulation of autophagy.

## 3 EXOSOMES DERIVED FROM MSCS MITIGATE VARIOUS DISEASES BY REGULATING AUTOPHAGY

MSCs, stem cells with the potential for self‐renewal and multi‐directional differentiation,^57^ produce various bioactive substances that can regulate immunity, activate endogenous stem/progenitor cells, and promote tissue repair, angiogenesis and anti‐apoptosis.^58^ However, their therapeutic properties are primarily attributed to paracrine effects, and studies have shown that components secreted by MSCs to the outside, such as exosomes, can have the same effect as cell transplantation. Furthermore, MSC‐derived exosomes (MSC‐Exos) have a lower immune rejection rate and are therefore more suitable for therapeutic use, compared with MSCs.^59^ In addition, MSC transplantation is an effective treatment strategy in regenerative medicine to repair injured organs via the regulation of autophagy. At present, an increasing number of studies have shown that MSCs can improve various diseases by producing exosomes, which transfer their cargos to recipient cells and regulate autophagy.

### 3.1 Cardiovascular disease

MSC‐Exos are widely involved in the development of cardiovascular diseases via the regulation of autophagy. Xiao et al^60^ found that post MI, miR‐125b‐5p in MSC‐Exos inhibited ischaemia‐induced autophagy and improved cardiac function by targeting the p53/Bnip3 signalling pathway. Jiang et al^61^ treated H9c2 cells with I/R, and found that the apoptosis and autophagy induced by MSC‐Exos were reduced following intervention. Meanwhile, Liu et al^62^ found that hypoxic preconditioning increased the secretion of exosomes from human umbilical cord MSCs (hucMSC‐Exos), which has been found to inhibit autophagy through the PI3K/Akt/mTOR pathway, thereby inhibiting the apoptosis of H9C2 cells induced by hypoxia and serum deprivation. Li et al^63^ demonstrated that exosomal miR‐301 secreted by bone marrow MSCs (BMSCs) protected MI tissues by inhibiting myocardial autophagy, whereas Rajshekhar et al^64^ found that cultured rat cardiomyocytes exposed to lipopolysaccharide (LPS) increased cardiomyocyte apoptosis. MSC‐Exos inhibited the apoptosis and autophagy of cardiomyocytes, thus promoting cell survival. Gong^65^ discovered that exosomes derived from BMSCs overexpressing stromal‐derived factor 1 inhibited autophagy of ischaemic cardiomyocytes and promoted micro‐angiogenesis of endothelial cells. However, other studies have suggested that MSC‐Exos can relieve MI by inducing autophagy. Liu et al^66^ illustrated that exosomes derived from BMSCs enhanced autophagy and reduced the apoptosis of H9C2 cells stimulated by H_2_O_2_ in vivo and in vitro. Furthermore, authors demonstrated that the above effects were partly mediated by AMPK/mTOR and Akt/mTOR signals. Consistent with these findings, Michelle et al^67^ also demonstrated that induced pluripotent stem cell‐derived cardiomyocyte (ICM)‐derived exosomes (ICM‐Exos) improved cardiac function following MI by upregulating autophagy in hypoxic cardiomyocytes. Researchers have reported a beneficial effect from myocardial autophagy activation in the early stage of MI; however, during the reperfusion stage, excessive autophagy could lead to cell death.^68^ MSC‐Exos can protect the myocardium by activating moderate autophagy or inhibiting excessive autophagy, whereas the specific mechanisms and signalling pathways depend on the establishment of the myocardial model and the type of MSCs. In addition, exosomes derived from umbilical cord MSCs (UCMSCs) reportedly alleviated viral myocarditis by activating the AMPK/mTOR‐mediated autophagy flux pathway.^69^

### 3.2 Nervous system disease

MSC‐Exos can improve cerebral ischaemic injury and PD by regulating autophagy. Huang et al^70^ established a model of middle cerebral artery occlusion and reperfusion in male SD rats and confirmed that exosomes from pigment epithelium‐derived factor‐modified adipose‐derived MSCs improved brain I/R injury by activating autophagy and inhibiting neuronal apoptosis. Jiang et al^71^ demonstrated that exosomes loaded with miR‐30d‐5p reversed ischaemia‐induced autophagy and brain injury by promoting M2 microglia/macrophage polarization. Chen et al^72^ found that hucMSC‐derived exosomes had a therapeutic effect on SY5Y cells and SD rats exposed to 6‐OHDA, and that autophagy played an important role in mediating the beneficial effect of exosomes on PD.

### 3.3 Liver diseases

MSC‐Exos can improve hepatic fibrosis, hepatic ischaemic injury and liver failure by regulating autophagy. Qu et al^73^ found that exosomes containing miR‐181‐5p increased autophagy and alleviated TGF‐β 1‐induced liver fibrosis by inhibiting the STAT3/Bcl‐2/Beclin‐1 pathway in HST cells and CCl4‐induced liver fibrosis in mice. Yang et al^74^ demonstrated that exosomes derived from BMSCs effectively enhanced autophagy and reduced liver I/R injury both in vivo and in vitro. Exosomal miR‐20 derived from UCMSCs reduced liver I/R injury by inhibiting Beclin‐1‐mediated autophagy.^75^ Similar to the role in cardiac I/R, autophagy also plays a dual role during hepatic I/R; that is, it plays a protective role during the early stage of hepatic I/R and a deleterious role during the reperfusion stage.^76^ MSC‐Exos protect the liver by activating moderate autophagy or inhibiting excessive autophagy in different stage of hepatic I/R. In addition, BMSC‐Exos can reduce hepatocyte apoptosis following acute liver failure by promoting autophagy.^77^

### 3.4 Kidney disease

MSC‐Exos can improve diabetic nephropathy (DN) and renal injury by regulating autophagy. Ebrahim et al^78^ found that MSC‐Exos induced autophagy through the mTOR pathway to mitigate DN. Consistent with these findings, He et al^79^ found that exosomes secreted from adipose‐derived stem cells (ADSCs) enhanced autophagy flux and reduced podocyte injury in spontaneous diabetic mice by inhibiting the activation of the mTOR signalling pathway. Furthermore, the authors also found that miR‐486 was the key factor in ADSCs and played a crucial role in improving DN symptoms both in vivo and in vitro. Wand et al^80^ found that autophagy was induced in rat renal epithelial cells co‐cultured with hucMSC‐Exos via the transportation of 14‐3‐3ζ protein, thus protecting HK‐2 cells from cisplatin‐induced damage. Further results confirmed that 14‐3‐3ζ promoted the formation of autophagosomes through ATG16L interaction.^81^

### 3.5 Other diseases

In addition to the mentioned diseases above, MSC‐Exos can also improve chronic obstructive pulmonary disease, osteoarthritis (OA), retinal detachment (RD) and spinal cord injury by regulating autophagy. Cigarette smoke can induce the upregulation of miR‐210 in bronchial epithelial cells, which inhibits autophagy by targeting Atg7, reduces myofibroblast differentiation, and improves pulmonary fibrosis.^21^ Wu et al^82^ found that infrapatellar fat pad (IPFP) MSC‐derived exosomes partly enhanced the autophagic level of chondrocytes by inhibiting mTOR, and protected against articular cartilage from injury in OA mice. The underlying mechanism may be associated with the inhibition of the mTOR pathway by exosomal miR100‐5p. In RD, MSC‐Exos inhibited the apoptosis of photoreceptor cells and maintained normal retinal structure by inhibiting autophagy.^83^ Gu et al^84^ reported that BMSC‐Exos reduced neuronal apoptosis and promoted functional behaviour recovery by promoting autophagy in rats with spinal cord injury. Although MSC‐Exos can alleviate various diseases, they may also promote tumorigenesis. Huang et al^85^ illustrated that human BMSC‐Exos promoted tumorigenesis and metastasis by inducing carcinogenic autophagy in osteosarcoma.

## 4 REGULATION OF EXOSOME BIOGENESIS AND RELEASE VIA AUTOPHAGY

As shown above, extracellular exosomes can regulate the intracellular autophagic processes through various signalling pathways, and play an important role in various diseases. In turn, evidence indicates that autophagy can regulate the biogenesis and release of exosomes in cells, which has implications in both physiological and pathological implications.

### 4.1 Under physiological implications

**FIGURE 4:**
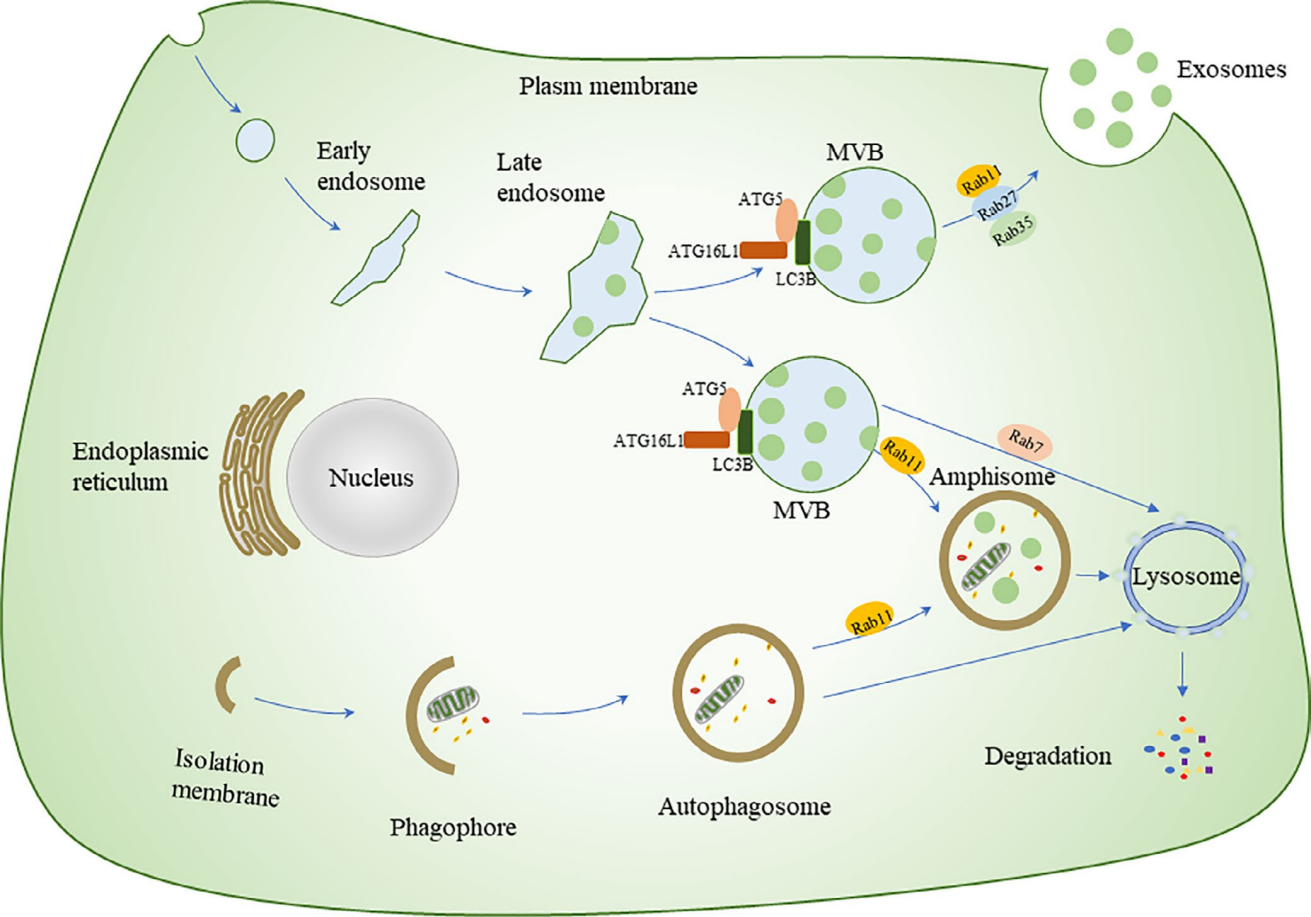
Crosstalk between exosome release and the autophagic process. Autophagy can regulate the release of exosomes. As shown in the figure, the amphisomes represent the intersection of exosome biogenesis and the autophagic process. Different autophagic‐related proteins (ATG5, ATG16L1 and LC3B) located on the membrane of MVBs, facilitate exosome biogenesis. Rab7 mediates the transport of MVBS to lysosomes. Rab11 promotes the fusion of MVB and autophagosomes into amphisomes. Rab11, Rab27 and Rab35 are involved in MVBs fusion with the PM to release exosomes. PM, plasma membrane; MVB, multiple vesicle bodies

The secretory process of exosomes is complex, yet orderly. First, with the help of the endosomal sorting complex required for transport (ESCRT) complex, the plasma membrane (PM) is trapped in the early endosome and late endosome, and further trapped in multiple vesicle bodies (MVBs), which have two different fates: (a) fuse with autophagosomes to form amphisomes, which are transported to lysosomes for degradation, or fuse with lysosomes for degradation directly; (b) fuse with the PM and be secreted outside the cell to form exosomes.^6^ Growing evidence suggests that autophagy‐related proteins contribute to exosome biogenesis. ATG5, ATG16L1 and LC3B, locate on the MVB membrane, have a curtail role in exosome biogenesis. The ATG5‐ATG16 L1 complex also facilitates the dissociation of V‐ATPase, preventing the acidification of MVBs and their subsequent lysosomal degradation. MVBs then fuse with the PM to release exosomes.^86^ In addition, Rab family proteins participate in the formation of autophagy, whereas various Rab‐GTPase proteins (including Rab7, Rab11, Rab27 and Rab35) are involved in the intracellular trafficking of MVBs/exosomes. In the degradation pathway, Rab7 mediates the transport of MVBs to lysosomes and regulates the energy balance by hydrolysing the MVBs cargo.^87^ Rab11 promotes the fusion of MVB and autophagosomes into amphisomes. Rab11, Rab27 and Rab35 are involved in MVBs fusion with the PM to release exosomes into the extracellular milieu^88^ (Figure 4). The coordination of these pathways helps to maintain intracellular homeostasis and is affected by specific key molecules. Induced autophagy can promote the fusion of MVBs and autophagosomes, thereby inhibiting the release of exosomes from the erythroleukemia cell line K562.^87^ Murrow et al^89^ illustrated the importance of the ESCRT‐associated protein Alix was important in late endosome distribution and exosome biogenesis. Autophagy is inhibited by CAV1 to protect MVBs from fusing with autophagosomes, thereby promoting exosome secretion.^90^ Zhao et al^91^ found that exosome release, like autophagy, is negatively regulated by mTORC1 in response to changes in nutrition and growth factors. These findings are not consistent with the phenomenon that the release of exosomes is inversely proportional to autophagy activity. As is known, autophagy is negatively regulated by mTORC1. Recent studies suggested that mTORC1 acts during the late stage of exosome biogenesis, likely at the stage of the docking/fusion of MVBs with the PM. mTORC1 controls exosome release in a Rab27A‐dependent matter. The concurrent regulation of autophagy and exosomes by mTORC1 allows cells to coordinate waste management and recycling, which is likely to play a key role in cell fitness under various adverse conditions.^91^

### 4.2 Under pathological implications

Malignant diseases such as neurodegeneration, cancer and microbial infection can develop with insufficient autophagy and the increased release of exosomes. Insufficient autophagy limits the clearance of intracellular α‐synuclein, which aggravated the progression of PD.^14^ Fussi et al^92^ further discovered that silencing ATG5 inhibited the formation of autophagosomes and increased the extracellular secretion of α‐synuclein. Although the increase in autophagy tends to reduce the release of exosomes, autophagy and exosomes may increase or decrease at the same time under certain conditions. Shrivastava et al^93^ found that inhibiting the autophagy pathway impairs the exosome‐mediated release of infectious hepatitis C virus (HCV) particles. The authors also observed that knockdown of Rab27a inhibits the generation of intracellular and extracellular infectious HCV particles. Oxidative stress can increase autophagy of retinal astrocytes (RAC) and further promote RACs to damage endothelial cell function by releasing exosomes.^94^ Inhibition of autophagy or exosomes was shown to reverse this injury, although the mechanism remains unclear.

## 5 ROLE OF AUTOPHAGY‐REGULATING EXOSOMES IN DELAYING THE DEVELOPMENT OF DISEASES

Under pathological conditions, abnormal autophagy can cause the release of harmful exosomes and accelerate disease progression. Reducing the release of harmful exosomes could alleviate tissue damage, especially in liver diseases. For example, the increased expression of miR‐155 induced by alcohol increases the release of harmful exosomes by targeting mTOR in the autophagic pathway. Silencing miR‐155 restored autophagy and reduced exosome release, which may curtail the occurrence of alcoholic liver disease.^95^ Autophagy impairment mediated by TRIB3 and the selective autophagy receptor SQSTM1 in human liver fibrosis promoted the secretion of harmful exosomes, which induced the migration, proliferation and activation of HSCs. Disrupting the TRIB3‐SQSTM1 interaction reduced liver fibrosis by restoring autophagy and inhibiting exosome‐mediated HSC activation.^96^

## 6 CONCLUSIONS AND PERSPECTIVES

Generally, exosomes and autophagy are closely associated. In this review, we highlighted new ideas for disease prevention by identifying key signalling pathways and targets with mutual influence. Although exosomes exist in extracellular fluid (primarily blood), they can release their cargos into target cells via endocytosis, receptor ligand binding and membrane fusion. These cargos can regulate the autophagic level of target cells through the mTOR and Beclin‐1 signalling pathways, and autophagy‐related proteins, thus affecting the development of associated diseases. MSC‐Exos can mitigate diseases by regulating autophagy, especially in cerebral ischaemia, MI, liver fibrosis, and DN. In the future, we will explore the therapeutic role of MSC‐Exos and autophagy in other diseases. In addition, intracellular autophagy can affect the biogenesis and release of exosomes to maintain intracellular homeostasis under physiological conditions. Abnormal autophagy can lead to the increased release of harmful exosomes, whose resulting damage can be attenuated by interfering with the autophagic process. However, promising exosomal and autophagic research is largely conducted using cell culture systems, which requires further validation using animal models and physiology‐related experimental conditions.

## CONFLICT OF INTEREST

The authors declare no conflict of interest.

## AUTHOR CONTRIBUTIONS

**Huifang Xing:** Conceptualization (lead); Writing‐original draft (supporting); Writing‐review & editing (equal). **Jin Tan:** Conceptualization (equal); Writing‐original draft (supporting); Writing‐review & editing (supporting). **YuYang Miao:** Writing‐review & editing (equal). **Yingmei Lv:** Writing‐review & editing (equal). **Qiang Zhang:** Conceptualization (supporting); Funding acquisition (lead).

## Data Availability

I confirm that I have included a citation for available data in my references section.
